# Venous Thromboembolism in Sepsis: From Bench to Bedside

**DOI:** 10.3390/biomedicines10071651

**Published:** 2022-07-08

**Authors:** Eleonora Galli, Elena Maggio, Fulvio Pomero

**Affiliations:** 1 Internal Medicine Residency Program, University of Turin, 10100 Turin, TO, Italy; eleonora.galli@unito.it; 2 Department of Internal Medicine, M. and P. Ferrero Hospital, 12060 Verduno, CN, Italy; emaggio@aslcn2.it

**Keywords:** sepsis, thromboembolism, venous thrombosis, inflammation, thromboinflammation

## Abstract

Septic patients were commonly affected by coagulation disorders; thus, they are at high risk of thrombotic complications. In the last decades, novel knowledge has emerged about the interconnected and reciprocal influence of immune and coagulation systems. This phenomenon is called immunothrombosis, and it indicates an effective response whereby immune cells and the coagulation cascade cooperate to limit pathogen invasion and endothelial damage. When this network becomes dysregulated due to a systemic inflammatory activation, as occurs during sepsis, it can result in pathological thrombosis. Endothelium, platelets and neutrophils are the main characters involved in this process, together with the TF and coagulation cascade, playing a critical role in both the host defense and in thrombogenesis. A deeper understanding of this relationship may allow us to answer the growing need for clinical instruments to establish the thrombotic risk and treatments that consider more the connection between coagulation and inflammation. Heparin remains the principal therapeutical response to this phenomenon, although not sufficiently effective. To date, no other significant alternatives have been found yet. In this review, we discuss the role of sepsis-related inflammation in the development and resolution of venous thromboembolism and its clinical implications, from bench to bedside.

## 1. Introduction

Thrombosis is the pathophysiological mechanism underlying the three main cardiovascular disorders [1], and of these, venous thromboembolism (VTE) is the third-most common cause of cardiovascular mortality, after myocardial infarction and stroke [2], with the annual incidence rate ranging from 0.75 to 2.69 per 1000 subjects, which increases between 2 and 7 per 1000 among people aged ≥70 years [1]. Moreover, in high-income countries, VTE associated with hospitalization is the second-most common cause of disability-adjusted life years (DALYs) lost [3], with an impact greater than nosocomial pneumonia, catheter-related bloodstream infections and adverse drug events. Therefore, venous thromboembolism is an important contributor of the global burden of disease [1], as well as sepsis, that presents an incidence and mortality rate, respectively, of about 48.9 million incident cases worldwide and 11.0 million sepsis-related deaths in 2017 [4], often with long-term effects in terms of physical, psychological, and cognitive morbidity that impact on healthcare with important social implications [5,6], being one of the most costly inpatient conditions with a constantly increased trend and accounting for more than USD 38 billion in the USA [7].

Sepsis is a life-threatening syndrome characterized by multi-organ dysfunction, consequent to a dysregulated immune, endocrine and metabolic response to infection [8,9] and clinically codified using the Sequential Organ Failure Assessment (SOFA) score [10]. Septic patients are generally affected by coagulation disorders [11]; thus, they are at a high risk of thrombotic complications, ranging from widespread microvascular involvement, such as disseminated intravascular coagulation (DIC), to venous thromboembolism, arising as deep vein thrombosis (DVT) or pulmonary embolism (PE) [12,13]. 

Historically, Virchow’s triad was the cornerstone of the knowledge about pathophysiological processes leading to thrombosis; it consists of three elements strictly interconnected with each other: blood flow alterations, vascular wall damage or dysfunction and hypercoagulability [14,15]. Despite the impact of the triad on our understanding of venous thromboembolism, it is no longer sufficient alone to explain the complex mechanisms that produce a thrombotic event and, in particular, stasis; related to bed rest and hospitalization, which is a known risk factor for VTE, it is usually not enough to lead to thrombosis. From the pioneering theories of Gwendylen Stewart in the early 1970s, over the years, a growing body of evidence has suggested the role of inflammation in the pathophysiology of VTE [15], highlighting a correlation between venous thrombosis and inflammatory disorders, such as sepsis, adding contextual elements to the classical Virchow’s triad.

During sepsis, several pathophysiological processes occur simultaneously [11], and among them, the dynamic relationship between coagulation and inflammation assumes much more relevance. In the past, many working groups tried to define and describe this relationship: the first one was Tanguay J-F et al. in 2004 that used the term “thromboinflammation” to explain the platelet–leukocyte interaction through P-selectin and P-selectin glycoprotein ligand 1 (PSGL-1) [16]; then, in 2009, Blair P. et al. used it to indicate the stimulation of platelets through Toll-like receptor 2 (TLR2) [17]. In 2013, the term “immunothrombosis” was coined by Engelmann and Massberg [18] to explain this complex and reciprocal interaction, whereby, on the one hand, the activation of a coagulation cascade triggers the immune system, cooperating with the identification, containment and destruction of pathogens [18], and on the other hand, the innate immune cells promote the development of thrombus [12,14].

In this context, many actors are involved, starting from endothelium, platelets and coagulation factors, cytokines and immune cells to their complex interactions, that appear to be at the basis of not only the microvascular phenomena but also of deep vein thrombosis, depending on the site, depth and extent of vascular wall damage and, consequently, on the specific activated pathway [19,20]. Interestingly, despite a deeper understanding of the pathophysiology underlying VTE in sepsis and the strict relationship between these two conditions, the current therapy available for VTE consists of prophylactic or therapeutic strategies targeting coagulation factors without taking into account the inflammatory processes that lead to thrombosis, leaving a significant gap in the effective treatments and prevention of VTE.

Therefore, the purpose of this review is to collect and summarize the current knowledge on the subject by investigating the molecular and immunologic pathways of venous thrombosis and underlining the role of sepsis-related inflammation in the development and resolution of VTE and their clinical implications.

## 2. Materials and Methods

First of all, we identified some key words to explore the association between sepsis and venous thromboembolism, such as sepsis, thromboembolism, venous thrombosis, deep vein thrombosis, thromboinflammation and coagulopathy. Then, we performed a literature search in the PubMed and Medline databases from 1 January 2012 to 31 January 2022, examining in depth the various characters of the process and the relationship between them. We favored the inclusion of articles from the last 10 years to give up-to-date information, although we did not exclude older reports when highly referenced and when more recent evidence was unavailable. Moreover, we also included some studies related to COVID-19 infection, which presents a particular elevated incidence of VTE, although the pathophysiological mechanisms are not fully understood yet. We selected the relevant references identified by the search strategy, and we excluded some of them if the title and/or abstract were not appropriate for the aim of the review. Full texts were obtained for relevant references and when the significance of an article could not be excluded from the abstract. We chose to consider this lapse of time and all types of articles to obtain a comprehensive overview of this topic, initially without any language restriction. Moreover, we supplemented our search by manually reviewing the reference lists of all the retrieved articles, not to exclude any relevant source of information.

## 3. From Bench

As it is well-known, the main purpose of hemostasis is to stop bleeding by repairing the damaged vascular wall after injury, with a series of highly regulated steps leading to the development of thrombin and fibrin. Furthermore, hemostasis has another protective function, phylogenetically ancient and evolutionarily preserved [21], aimed at containing and eliminating pathogens through the formation of local thrombosis. In both cases, endothelial cells, leukocytes and platelets are the primary cellular inputs engaged to ensure the inflammatory and immune responses and the activation of the coagulation factors. Immunothrombosis is the result of the reciprocal interaction between immune and coagulation systems after a pathogen’s entry into the bloodstream [18]. An exaggerated activation of these two systems, which can occur during sepsis, can lead microvascular and macrovascular thrombosis. 

### 3.1. The Role of Endothelium

The endothelium is an important regulator of vascular homeostasis with several physiological functions. It is a regulator of the vasomotor tone, a carrier for cells, nutrients and inflammatory/anti-inflammatory signaling; it has natural anticoagulant properties through its glycocalyx made of heparan sulfates and chondroitin sulfate and through transmembrane molecules such as TM (thrombomodulin), EPCR (endothelial cell protein C receptor) and TFPI (tissue factor pathway inhibitor) [22]; it influences fibrinolysis by producing tPA (tissue-type plasminogen activator) [23] and uPA (urokinase plasminogen activator) [24]; at last, it is a barrier from bacterial invasion [25]. 

During sepsis, it undergoes various phenomena, including damage, activation and dysfunction, and acquires pro-adhesive, procoagulant and antifibrinolytic phenotypes, acting as a bridge between the immune system response and coagulation cascade. This phenotypic shift can be triggered by hypoxia, tissue injury, cytokine signals, hemodynamic changes, endotoxemia or pathogen recognition [22,26].

The first element of connection between the endothelium, immune cells and platelets during sepsis is related to “endothelial activation”, which leads to an increased expression and release of adhesion molecules with a pro-adhesive behavior. It is mediated by selectins expressed on ECs (E-selectin), on leucocytes (L-selectin) and platelets (P-selectin), by immunoglobulin-like receptors and receptors from the integrin family that form three heterodimers of β2-integrin collectively named the CD11/CD18 complex and, third, by intracellular adhesion molecules (ICAMs) that mediate the cross-talk with activated leukocytes [26]. In early endothelial activation, upon exposure to infectious and inflammatory agents, exocytosis of WPB (Weibel-Palade bodies) rapidly occurs and P-selectin and vWF (von Willebrand factor), contained in these cytoplasmatic preformed granules with molecules such as IL-6, IL-8, tPA (tissue plasminogen activator), etc., are delivered to the plasma membrane of endothelial cells [27,28]. Thus, vWF promotes platelet aggregation, binding them via the GPIbα (glycoprotein Ibα) receptor [29], and leukocyte recruitment is mediated by P-selectin via PSGL (P-selectin glycoprotein ligand-1) and by β2 integrins, with leucocyte extravasation because of the endothelial increased permeability [30]. The impact of the excessive expression of these molecules in septic processes is underlined by numerous experimental studies on animal models, whereby the use of their inhibitors or mice knockout for some of them presented better endpoints in terms of survival [31,32,33]. 

The regulation of expression, binding avidity and modulation of these adhesion molecules are operated by biological mediators such as circulating pathogen-associated molecular pattern (PAMPs) and damage-associated molecular pattern (DAMPs) molecules; endothelial-derived cytokines (IL-8, IL-1 and tumor necrosis factor α (TNFα)); local synthesis of the platelet activating factor (PAF); triggering intracellular signaling cascades that activate the transcription factors NF-κB (nuclear factor kappa light-chain enhancer of activated B cells) [34] and promoting neutrophil–endothelial adhesion and then neutrophils-activated platelet interactions [26].

**Figure 1:**
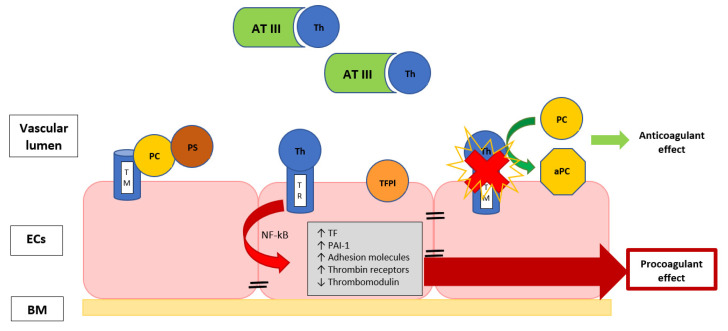
The image describes the EC surface with thrombomodulin, a thrombin-binding protein that is responsible for thrombin activity inhibition. When thrombin is bound to TM, they form an activator complex for PC, conferring the anticoagulant properties. The exposure to inflammatory and/or septic stimuli rapidly causes the internalization of TM or the release of inactivated TM, favoring the thrombin binding with its receptor, leading to the endothelial modulation ability towards a procoagulant state. Th, thrombin; TR, thrombin receptor; TM, thrombomodulin; AT III, antithrombin III; TFPI, tissue factor pathway inhibitor; NF-κB, nuclear factor-κB; PAI-1, plasminogen activator inhibitor; TF, tissue factor; BM, basal membrane; ECs, endothelial cells; PC, protein C; aPC, activated protein C; PS, protein S.

Furthermore, the endothelium can assume a procoagulant phenotype when it is damaged during sepsis. Vessel injury consists of cellular alteration or the interruption of the endothelial line, and it produces hypoxia and inflammation, provoking a sort of endotheliopathy with impairment of the membrane anticoagulant components, such as endothelial glycocalyx, tissue factor pathway inhibitor, thrombomodulin (TM) and protein C, with the induction of a hypercoagulability (Figure 1) [14,35]. 

This impairment assumes systemic characteristics because of the nature of disseminated sepsis that leads to generalized endothelial involvement [19] due to a diffuse host inflammatory reaction to the invading organism with the onset of a systemic procoagulant state [36]. In the past, some studies demonstrated that septic shock can affect the endothelium with anatomical injury [37,38,39], and experimental endotoxin-induced septic shock rapidly damages several endothelial cell functions [40,41,42]. For example, in rabbits, the infusion of lipopolysaccharide (LPS), an endotoxin on the membrane of Gram-negative bacteria, especially Escherichia Coli, induces prolonged endothelium-dependent vascular dysfunction [39], and in just 15 min from the inoculation, it causes the impairment of the major anticoagulant mechanisms [43]. 

There are many mechanisms involved in endothelial damage during sepsis, as shown in Table 1; they especially cause apoptosis and increased permeability, contributing to thrombosis and organ dysfunction. 

The endothelium of the venous system is more easily exposed to insults and more predisposed to the formation of macrothrombi in correspondence with the vein valve sinus, because in these areas, there is a condition of hypoxia and slowing of the blood flow [44]. It maintains a compensatory environment carefully regulated to prevent thrombosis, but the presence of an inflammatory setting, as in sepsis, can abolish this delicate antithrombotic balance in the valve sinus, removing natural anticoagulants and resulting in venous thrombosis [44,45].

### 3.2. The Role of Coagulation Cascade

In this procoagulant state begun from vascular wall injury, we can identify two main coagulation pathways that lead to thrombosis, triggered by a different depth of damage [46]. 

**Figure 2:**
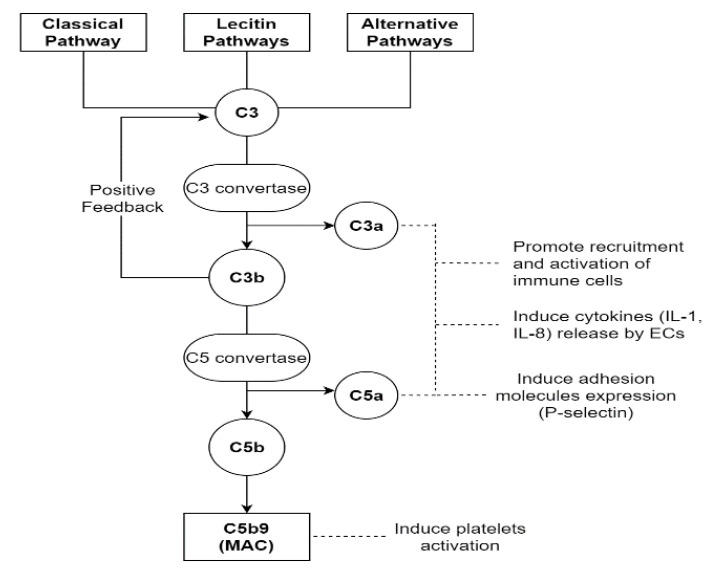
This figure shows the complement cascade starting from the classical, lectin and alternative pathways. All three lanes converge at the cleavage of the C3 element by their respective C3 convertases. This leads to MAC formation and consequent osmotic lysis of the pathogens. Moreover, C3a and C5a, as well as MAC, perform other functions, becoming another point of convergence for the immune and coagulation systems. MAC, membrane attack complex; ECs, endothelial cells.

On the one hand, if the injury due to complement activation is confined to the endothelium, mainly the von Willebrand factor (vWF) path is stimulated [19,47]. The activation of a complement cascade belongs to the innate immune response, and normally, it is a defensive mechanism against infectious microorganisms or endotoxins. It can occur through three different pathways: the classical (CP), the lectin (LP) or the alternative (AP) ones. The first two are triggered by the binding of C1q for CP and mannose-binding lectin for LP to DAMPs or PAMPs, respectively. The third one, through its own C3 convertase, presents a low level of constitutive activation, considered a sort of immune surveillance [48]. After the first contact with noxious agents, the serine proteases associated with C1q and mannose-binding lectin provoke the cleavage of C4 and C2 with the formation of their “a” and “b” split products [49]. This leads to a first opsonization of foreign surfaces by C4b, allowing the identification and elimination of invading pathogens by phagocytosis. Successively, C4b binds C2a, forming the C3 convertase, which splits C3 in C3a and C3b. C3b participates in opsonization too, and it amplifies complement activation because of a positive feedback loop. Furthermore, when its levels reach a critical threshold, it binds C4b2a with the formation of the C5 convertase (C4b2a3b), carrying on the complement cascade. Instead, C3a and C5a mediate other inflammatory and prothrombotic actions; in fact, they can induce endothelial expressions of cytokines (IL-1 and IL-8) through their G-protein-coupled receptors, with immune cell direct and indirect activation [50]. Moreover, C5a can promote the exposure of adhesion molecules such as P-selectin [51]. Instead, C5b allows the creation of the membrane attack complex (MAC), a multi-protein pore complex made of C5b-9, which causes the osmotic lysis of pathogens [52]. Despite this protective function, the MAC can also have a destructive action on the host endothelium, impacting the course of sepsis with endothelial dysfunction [19], which enhances the endothelial activation linked to the infection itself. The complement system is not only shown to have an effect on the endothelium, but it can also directly induce platelet activation and aggregation, as well as potentiate thrombin-induced platelet secretion and aggregation, stimulating the tissue factor expression on monocytes through C5b-7 action [13,52] (Figure 2). 

However, consequently to the isolated endothelial damage, an increased expression of surface adhesion molecules such as P-selectin and of vWF facilitates the subsequent binding of circulating platelets and leukocytes [14,53], leading to the formation of a vWF–platelets complex with thrombocytopenia and, predominantly, microvascular thrombosis [19].

On the other hand, in systemic inflammatory syndromes such as sepsis; chemical or physical vascular damage sustained by infectious pathogens; inflammatory cytokines (TNFα, IL-1 and IL-6); reactive oxygen species (ROS) or other injurious agents, can interrupt the integrity of the endothelial barrier with the exposure of collagen and tissue factor (TF) to the bloodstream, with TF rapid upregulation on perivascular cells and monocytes [54]. Tissue factor is a transmembrane glycoprotein belonging to the cytokine receptor superfamily, and the first effect of its exposure is to trigger the extrinsic coagulation cascade through the activation of Factor VII; moreover, the exposed TF ties a small amount of circulating Factor VIIa [55], already activated, and this link enhances the catalytic power of Factor VIIa, leading primarily to Factor X activation and thrombin generation and, in addition, to Factor IX activation, also involving the intrinsic coagulation pathways.

**Figure 3:**
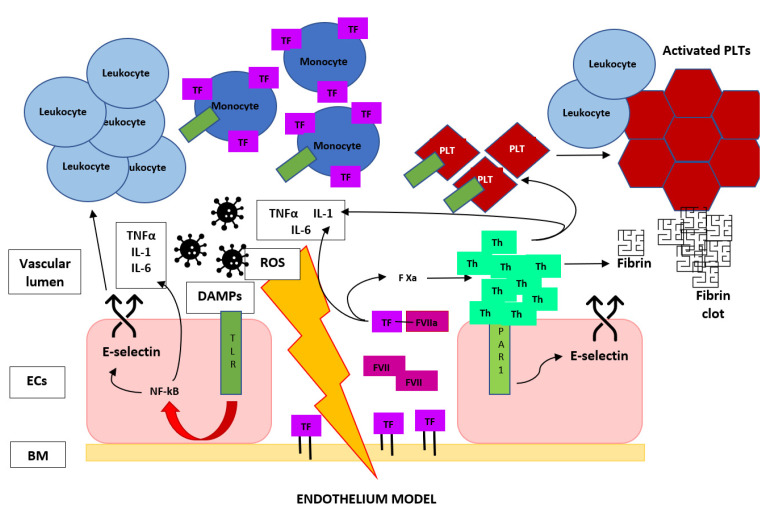
Brief graphic description of the escalating circle of events that start from vascular wall damage and lead to the crosstalk between the coagulation cascade and immune system in a potent positive feedback loop toward venous thrombus formation during sepsis, more underlying the essential role of TF and thrombin in DVT. TF, tissue factor; F VII(a), (activated) factor VII; F Xa, activated factor X; Th, thrombin; PLT, platelet; PAR-1, protease-activated receptor 1; TLR, Toll-like receptor; DAMPs, damage-associated molecular patterns; ROS, reactive oxygen species; ECs, endothelial cells and BM, basal membrane.

For this reason, many authors consider TF expression the initiating event in the coagulopathy of acute sepsis because of its central role in the activation of the coagulation cascade and proinflammatory pathways. In fact, through the protease-activated receptor (PAR) signaling, the TF VIIa complex induces the release of cytokines, chemokines and grow factors [56], and in turn, these elements directly mediate a natural anticoagulant suppression; induce the expression of additional TF, fibrinogen, factor VIII and von Willebrand factor—in particular, for the action of IL-6—and sustain vascular damage, promoting this procoagulant state [57]. This process is fueled by other DAMPs that promotes, through Toll-like receptor (TLR) signaling, the release of other cytokines, the expression of TF [58,59] and the induction of adhesion molecules on endothelial cells, which results in the recruitment and activation of leukocytes [60], with a further upregulation of TF on monocytes, entering in a positive feedback loop (Figure 3). The importance of TF in sepsis-related VTE is supported by the rapid and prolonged increase of tissue factor expression in septic animal models [39] and in septic patients and by the findings that reduced the levels of TF or lacking of the transmembrane domain of TF seemed to prevent coagulation abnormalities, excessive inflammation and organ dysfunction, with a prolonged survival in animal models [54,61,62].

The other TF effects are carried out by a second mediator mentioned above: thrombin. It is a serine protease that it is detectable within a few hours in sepsis experimental models obtained after the infusion of tumor necrosis factor and endotoxins [36,63,64]. Thrombin has a central role both in inflammation and coagulation cascades; in fact, on the inflammatory side, it mediates the activation of endothelial cells through PAR-1 signaling with the release of other proinflammatory cytokines and chemokines (monocyte chemoattractant protein-1, TNFα, IL-6, IL-8, IL-1 and PAI-1) and the induction of endothelial adhesion molecules (P- and E-selectin) [65,66], which results in the recruitment and activation of monocytes and neutrophils. These processes are known to occur during the early phase of venous thrombus formation during sepsis [67], as mentioned below, and they are configured as a point of convergence between inflammation and thrombogenesis. On the prothrombotic side, with its proteolytic action, thrombin changes fibrinogen into insoluble fibrin with fibrin clots formation [15,19], and then, it cleaves both PAR-1 and PAR-4 on the platelets’ surface, resulting in platelet activation [68,69,70], with consequent intracellular calcium mobilization, degranulation, morphological changes, the translocation of P-selectin and CD40 ligand on the surface and, finally, aggregation. 

Sepsis inflammation directly stimulates the release of platelet-activating factor (PAF), which accelerates platelets activation [71]. After P-selectin expression and consequent adhesion to leukocyte and endothelium [72,73], platelets contribute to the upregulation of TF and provide another surface for thrombin generation [74], enhanced by the discharge of granules containing Factor V, fibrinogen and Factor XIII. The aggregation mechanism is further amplified by the platelet release of thromboxane A2 and adenosine diphosphate, acting on closer platelets through thromboxane receptors and P2Y12 receptors, and it is stabilized by αIIbβ3 receptors on the platelet surface [68], allowing the binding of vWF and fibrinogen to support the rising aggregation. 

Ideally, the endothelium can oppose thrombin production when a vessel is repaired, balancing procoagulant and anticoagulant pathways after injury. However, when the local damage becomes systemic and sustained, as in sepsis, there is an upregulation of procoagulant mechanisms with a shift towards this state [36,75], and the vWF and TF pathways tend to converge, combining venous micro- and macrothrombosis [19,20], with a prominent role of TF in the development of VTE [48,76].

### 3.3. The Role of Leukocytes

As mentioned above, an increasing body of evidence supports an important role of immune cells not only in the response to pathogens but also in the genesis of thrombotic processes that occur during sepsis. A prominent role is played by monocyte for the exposure and release of TF [54]. However, recently, neutrophils have been gaining in importance; they are the most abundant leukocytes in humans [11] and the first line of defense during sepsis with the phagocytosis, degranulation and formation of neutrophil extracellular traps (NETs), on the one hand, to contain and eliminate infectious agents [77] and, on the other hand, to contribute to thrombogenesis. NETs are structures evolutionarily conserved [78] composed of cell-free DNA (cfDNA); histones (H2, H3 and H4), and neutrophil granule proteins, such as elastase, myeloperoxidase (MPO) and cathepsin G [12,14,77,79]. They are formed after stimulation with a variety of chemical stimulators (LPS, phorbol-12-myristate-13-acetate (PMA), IL-8, hydrogen peroxide and calcium ionophore); bacteria (S. aureus, S. suis, Salmonella enteric and K. pneumoniae) [77,79,80,81] and after interaction with platelets, connecting coagulation and immune systems and increasing the procoagulant activity of innate immune cells [12,18,67,82]. It should be noted that an infection accelerates neutrophil recruitment and activation, with a more pronounced involvement of NETs in venous, arterial and pulmonary thrombosis because of a defect to degrade NETs during severe bacterial infections [83].

Everything starts with the adhesion of monocytes and neutrophils to the venous wall through two main interactions: with the binding of PSGL-1 on the leukocyte surface to P-selectin exposed on activated endothelial cells and platelets [67] and with the connection of the endothelial CXC ligand with leukocyte CXCR2 (C-X-C motif chemokine receptor 2) [84]. 

Neutrophils participate in the sepsis procoagulant phenotype; first, with the degradation of natural anticoagulants through the direct TM cleavage by neutrophil elastase, MPO and serine proteases [26] and, second, with the release of TF-positive microparticles and highly charged molecules such as H3, H4 and DNA [85], with activation of the extrinsic pathway of the coagulation cascade, leading to activated factor X formation and thrombin production [14,53]. Histones and DNA complexes can also activate platelets through TLR-2 and TLR-4, induce direct cytotoxicity to endothelial cells and facilitate platelet–endothelium interactions [86]. Moreover, NETs provide a scaffold for platelets adhesion and activation, erythrocytes entrapment, fibrin polymerization and the accumulation of other procoagulant molecules, such as vWF, to promote thrombus formation and growth [79,87,88], also limiting fibrinolysis by tPA [88]. Some evidence demonstrates that NETs can be identified in human thrombi, whether arterial, venous or microvascular [67,89], and that they are increased in patients with venous thromboembolism [90,91], but their use as biomarkers for VTE is not yet established [14]. 

Extracellular trap (ET) production has also been described in other leukocytes such as monocytes, eosinophils and basophils. Monocyte ET release depends on oxidative burst, and also, in this case, it has been demonstrated as a procoagulant activity, but at the present state of knowledge, the main trap producers in venous thrombosis remain neutrophils [92]. 

The role of neutrophils in thrombus formation is further confirmed by numerous proof: their depletion and their inability of producing NETs due to deficiency in peptidyl-arginine deiminase 4 (PAD 4), and NET degradation by the administration of DNase or deficiency in P-selectin prevents DVT in murine models [35,67,93,94]. However, neutrophils contribute to thrombosis regardless of NETs by releasing damage-associated molecular patterns (DAMPs), cytokines and extracellular vesicles that result in interactions with platelets and endothelial cells [48], demonstrating a multifaceted role of neutrophils both in thrombosis and in inflammation.

Other observations suggest that NETs and their degradation products can also counterbalance procoagulant stimuli and promote thrombolysis, probably following temporal kinetics, depending on changes of the inflammatory setting during sepsis. For example, in vitro, extracellular DNAs downregulate prothrombotic functions of neutrophils and monocytes binding TLR9 receptors [95], cathepsin G and neutrophil elastase cleave PSGL-1 restricting neutrophil interactions with platelets and the endothelium [96]; they also degrade fibrin, and histone H2B serves as a receptor for plasminogen on monocyte surfaces [97]. In VTE episodes, neutrophils themselves show biphasic kinetics in their immune and prothrombotic actions [15]; in early sepsis, massive neutrophil activation contributes to lung and heart damage—in particular, after pulmonary embolism—whereas, during the late phase, neutrophils are actively involved in thrombosis resolution, as it can be seen from some studies conducted on animal models [98,99].

Considering the above, neutrophils appear as an interesting target for therapeutic interventions, but their systemic modulation or depletion would compromise the entire host defense system, so it is not a sustainable therapeutic option. Therefore, the attention is shifting on multiple strategies to inhibit NET effects as potential treatments for both uncontrolled inflammatory and thrombotic diseases [100].

### 3.4. COVID-19 Experience

As we learned from the last few years of a pandemic, the Coronavirus disease of 2019 (COVID-19) presents a significantly increased risk for micro- and macrovascular thrombosis, both in venous and arterial systems [101], with the incidence of DVT ranging from 13.7% and 14.8% and the incidence of PE about 16.5% [102,103], with a prevalence of about 27% and 32%, respectively [104], greater in critically ill patients rather than in non-ICU patients, due to multiple dysregulated molecular patterns as described above and well-reviewed specifically for COVID-19 by Higashikuni et al. [105] but common to various diseases, from SARS virus infections (including the SARS-CoV-2 one) to H1N1 influenza or bacterial sepsis. COVID-19 infection has demonstrated a greater and more prolonged inflammatory response than H1N1 influenza and bacterial sepsis. This seems to be not sufficient to fully justify the increased incidence and prevalence of thrombotic events [106], although histopathological findings have reported pulmonary microthrombi in 57% of COVID-19 and 58% of SARS patients, as compared with 24% of H1N1 influenza patients [107], with an increased angiogenesis and pulmonary microthrombosis, respectively, three and nine times more prevalent in patients affected by SARS-CoV-2 infection [108]. 

In COVID-19, the process seems to start from the downregulation of angiotensin converting enzyme 2 (ACE-2) activity due to its use by SARS-CoV-2 to enter human cells [109]. This enzyme is mainly localized on ciliated nasopharyngeal and lung cells but also on endothelial cells, heart, kidneys, testicles and brain [110], giving a first explanation about the primary site of infection and about the associated endothelitis. The inactivation of ACE-2 results in a reduced conversion of angiotensin II into angiotensin 1–7. This latter has important anti-inflammatory and antithrombotic functions [111] through the binding to MAS receptors on endothelial cells and through the production of nitric oxide (NO) and prostacyclin, which inhibits platelet activation and mediates an antioxidant action [112]. 

It is an excessive ROS production—in particular, from mitochondria—that triggers endothelial dysfunction. The resulting oxidative stress generates a positive feedback loop, which perpetuates the overproduction of ROS, mitochondrial dysfunction, endothelial damage and, consequently, a dysregulated inflammatory response, with chronic implications [113]. Upon endothelial injury, subendothelial vWF comes out, with further multimerization and the exposition of platelets and collagen-binding domains. Thus, vWF multimers act as a net that curbs platelets, facilitating their activation and aggregation with the consequent thrombosis [114]. In fact, during SARS-CoV-2 infection, a prominent role is played by platelets, which show an augmented activation and aggregation due to changes in the gene expression profile, partially attributed to multiple mitogen-activated protein kinase (MAPK) pathways activation and thromboxane generation [115]. This implies that patients with COVID-19 present a greater number of circulating aggregates of platelets with leukocytes, which enhances further platelet activation, TF activity, neutrophil extracellular traps formation and mitochondrial dysfunction, with a consequent worsening of the illness [89,101,116]. Platelets can also interact both with T and B cells; the first interaction is mediated by the major histocompatibility complex-1 (MHC-1), with antigen presentation and a consequent alteration of the cytokine release [115]; instead, the second interaction is made possible by the release and binding of platelet factor 4 (PF4), which leads to the maturation and clonal expansion of B cells. This occurs because the binding to glycosaminoglycans on the pathogen surface leads PF4 to a conformational modification with the exposure of a new epitope with the consequent attachment of antibodies; this PF4–antibodies complex acts not only on B cells but also employs phagocytes activity and enhances platelet activation in a positive self-amplifying loop [100,115]. The same mechanism of thrombosis mediated by the PF4–antibodies complex seems to occur in heparin-induced thrombotic thrombocytopenia (HITT) and in immune thrombotic thrombocytopenia due to some SARS-CoV-2 vaccines [100,117,118,119].

These mechanisms show also in COVID-19 the close interaction between an exaggerated immune response (sometimes autoimmune) and the activation of the entire coagulation system, but many aspects about the extent of the response, the thrombotic events and the impact of interindividual differences remain still unknown.

## 4. To Bedside

### 4.1. Diagnosis Approach

Coagulation disorders have a huge impact on patients’ prognosis, because they are a major cause of death during sepsis. They range from a slight decrease in platelet count to more dangerous clinical phenotypes very different from each other, such as VTE or DIC. Usually, coagulation dysfunctions are evaluated with some laboratory tests, of which the first four listed in Table 2 are the most commonly used in clinical practice for septic patients and patients with thromboembolic events, highlighting how coagulation and inflammation influence each other. Unfortunately, they have some limitations: on the one hand, they do not reflect the concept of a dysregulated host response as it occurs during sepsis [5], and on the other hand, plasma-based coagulation tests erase platelets contribution to thrombosis, do not reflect coagulation mechanisms in vivo and do not provide qualitative or functional information; some of them are not routinely available and have a high sensibility but a low specificity in detecting the thrombotic event [36]. 

Therefore, there is a growing need for clinical instruments that more consider the connection between coagulation and inflammation. On this line of thought, the Scientific and Standardization Committee (SSC) of the International Society on Thrombosis and Hemostasis (ISTH) defined both the ISTH overt DIC and sepsis-induced coagulopathy (SIC) scoring systems [120]. The standard methods used for the diagnosis of sepsis-induced coagulopathy are the platelets count, INR and SOFA score, but interestingly, some evidence is growing about the diagnostic role of thromboelastography [121,122], the viscoelastic measurements of whole blood able to evaluate the platelet function as well, even if studies on its role to guide therapy choice are lacking.

The diagnostic approach of VTE in septic patients starts from the evaluation of the usual clinical prediction rules, such as Wells and Geneva scores. However, it is not clear whether these scores show a reduced performance or not in septic patients who may present clinical characteristics similar to VTE, such as tachycardia, hypoxia and prolonged immobilization, to name a few. In the future, the diagnostic performance of the usual clinical prediction rules could be improved by adding the dosage of blood elements related to the intertwining of coagulation and immune systems. For example, soluble P-selectin associated with a Wells score has shown a specificity of 96% and a PPV of 100% in detecting VTE [123]. As said above, common laboratory tests used in the diagnosis of VTE are subject to imprecision, because the D-dimer is frequently altered due to factors related to infection. Instead, the instrumental approach maintains an important relevance, both in DTV and PE. In fact, in case of DVT ultrasonography is simple to apply also in the septic and unstable patient, because it can be used at the bedside, and it is easy to learn even by physicians not dedicated to vascular diagnostics [124]. It is important to remember that, in septic patients, the thrombotic phenomena can occur in uncommon sites, such as arterial ones, so in the diagnostic phase, it is decisive to keep in mind the differential diagnosis with these atypical presentations. For pulmonary embolism, echocardiography can be strongly suggestive, but diagnosis is made by performing a computer tomography (CT) with contrast medium. [125]. Often, in septic patients, this imaging method cannot be used due to sepsis-related acute kidney injury, unless the patient undergoes dialysis treatment afterwards. In these cases, alternative imaging tests are still advocated, and the use of magnetic resonance imaging (MRI) seems to give promising results, with a high specificity but a limited sensitivity [126].

Moreover, there is a lack in predictive instruments that consider also the influence of inflammatory processes—in particular, during sepsis. In general, for medical inpatients is important to be able to predict the thromboembolic risk in order to set the appropriate prophylaxis. Two scores are usually used for this purpose: the PADUA Prediction Score and the IMPROVE-DD (International Medical Prevention Registry on Venous Thromboembolism-D-dimer) VTE risk score. In septic patients, the first one is not so valuable; in fact, Vardi et al. found that the PADUA Prediction Score was insufficient to detect subjects likely to develop venous thromboembolism and ineffective in predicting anticoagulant administration. Nevertheless, it was capable of identifying the risk of poor outcome and mortality of these patients, working as a comorbid index rather than a specific VTE predictor [127]. Instead, recently, the IMPROVE-DD VTE risk score, which has already proven effective in predicting the thromboembolic risk in hospitalized medical ill patients [128,129], shows promising results as risk assessment model in patients affected by an infectious disease, such as SARS-CoV-2, with a sensibility of 97% and NPV about 99% but specificity of 21.5%, demonstrating a good discrimination of VTE risk [130]. 

Additionally, laboratory tests show analogue imprecision in septic patients, because they lose their diagnostic power according to their low specificity so that the results are altered due to the infection itself. A new element of interest could be the direct or indirect measurement of elements connected with the thromboinflammatory process, such as cell adhesion molecules or neutrophils products. For example, NETs or some of its components, such as MPO-DNA complexes, cfDNA, citrullinated histones H3 or neutrophil elastase (NE), contribute both to pathogens elimination and to thrombogenesis. In fact, on the one hand, sepsis is the predominant ICU condition associated with NET formation, and the levels of NETs are significantly related to disease severity [131]. On the other hand, NETs are closely associated to altered coagulation with thrombocytopenia, abnormal PT, aPTT, fibrinogen and D-dimer [11]. In fact, as described above, they influence the initiation, growth and resolution of DVT and can independently predict the development of thrombosis—in particular, in the form of DIC and mortality [131]. MPO is used to evaluate polymorphonuclear (PMN) cell family activation [15], and its activity reaches its peak 24 h after PE and then decreases until it returns to its basal level within seven days after PE [132]. Elevated levels of both circulating nucleosomes and NE/α1-antitrypsin complexes were associated with a three-fold risk of DVT, suggesting a dose-dependent relationship between the NET release and DTV [91]. CfDNAs provide a stimulus for clot formation, as well as trap pathogens [133], and their circulating levels, significantly elevated in septic patients [134], are associated with impaired fibrinolytic activity [12,133]. Moreover, high levels of cfDNA correlate positively with D-dimer, vWF and MPO [90] and show a discriminative power to predict mortality in septic patients [135]. Some recent evidence revealed an increase of NET circulating biomarkers in patients with SARS-CoV-2 infection dead from the disease and who developed PE; nevertheless, restricting the analysis to the most severe patients, the link between NET biomarker levels and survival is confirmed but not for PE occurrence [136]. Therefore, it has been suggested that early recognition of NETs may be a valuable biomarker of early sepsis [131], but other studies are need to established their role in defining thrombotic risk. 

### 4.2. Therapeutic Management

From the therapeutic point of view, the latest guidelines of Surviving Sepsis Compaign (SCC) recommend only VTE prophylaxis and the balance of overall effects favored low molecular weight heparin (LMWH) over unfractionated heparin (UFH) [9]. 

These drugs are administered for their anticoagulant properties, and it is not currently known if they have some influence on the inflammatory response caused by sepsis. In fact, the use of heparin and other anticoagulant agents do not take into account the reciprocal and close correlation between immune system and coagulation factors, acting mainly on these second ones. Nevertheless, some evidence has shown that post-treatment circulating levels of cfNDA, NE and the MPO–NDA complex in patients with early administration of heparin (within 6 hours) are significantly lower than those patients with late administration [12], suggesting a possible action in the early sepsis and therefore in the initial stages of endothelial damage. Furthermore, heparin can block the direct binding of vWF and cfDNA [137] and can reduce the expression of NETs, histones, and proinflammatory factors [138]. On the other hand, UFH and LMWH could be compromised by high affinity binding to circulating histones [139], and a large amount of heparin can produce antibodies against PF4 [140], an well as directly induce the formation of NETs, causing an excessive activation of coagulation [141] and immune response, leading to HIT [142]. 

Despite receiving a drug with anticoagulant properties, nearly 5–10% of people with sepsis and organ failure suffer of VTE [143,144], and routine pharmacologic prophylaxis appears underutilized [145]. Furthermore, the discussion about the best dosages of heparin to use is still open, because in some cases the anticoagulant dosage was more effective than the prophylactic one, in particular in patients at high risk of DIC, but this consideration is not universally accepted because of increased risk of bleeding and possible interference with bacteria clearance [146]. In this sense, the COVID-19 experience has so far demonstrated that in critically ill patients intermediate dose [147,148] nor anticoagulant dosages of UFH or LMWH do not lead to outcomes improvement in terms of a major probability of survival or a greater number of days free of cardiovascular or respiratory organ support [149]. Instead, there is some evidence of benefits of therapeutic anticoagulant dose of LMWH in preventing death or thrombosis in patients with a less severe form of COVID-19 [150], in particular if they present elevated levels of D-dimer [151,152], without a consistent increased risk of bleeding. Maybe, the use of fondaparinux could be an interesting alternative. It is a synthetic heparin-derived pentasaccharide designed to potentiate antithrombin-mediated inhibition of FXa but not of thrombin and accelerates antithrombin inactivation of FVIIa and FIXa [153,154]. Fondaparinux has major advantages over UFH including longer half-life and bioavailability, subcutis administration, easier dosing, and lack of HIT risk [155]. In some baboon models, fondaparinux shows protection against sepsis-induced DIC and inflammation and to provide survival benefit [154]. In particular, Keshari et al. found that fondaparinux treatment presented many positive effects: on the side of coagulation, it decreased the consumption of clotting factors, sustained the inhibition of both intrinsic and extrinsic pathways and inhibited the activation and amplification of coagulation cascade, without consumption of antithrombin; on inflammatory side, fondaparinux showed a better endothelial protection, a decreased E. coli-induced neutrophil degranulation and, at last, a substantial reduction of most of cytokines involved in septic inflammatory process [154]. These anti-inflammatory properties, in addiction to antiviral activities, emerged also during the COVID-19 pandemic, both for LMWH and fondaparinux [156], based on their role as a coadjuvant of serpins [157], accompanied with reductions in inflammatory markers during the period of treatment administration [158].

The major gap of VTE prophylaxis and therapy in septic patients is that the drugs commonly available are used mainly for their anticoagulant properties, without taking into account the pathophysiological mechanisms of immunothrombosis. The ideal therapy to prevent or treat VTE will suppress excess inflammation and coagulation without disrupting haemostasis or triggering immune deficiency [100]. Currently, some evidence is arising on the one hand about some mild anti-inflammatory properties of fondaparinux and LMWH, and on the other hand about the decrease of thrombosis with interventions targeting numerous inflammatory steps, both in vitro and in vivo models of DVT; these include the inhibition of cell adhesion molecules such as P-selectin [159,160], platelet activation [161], TF activity [162], NETs [163,164,165], cytokines and other coagulation factors [166].

A deeper and clearer understanding of the crosstalk between inflammation and coagulation can stimulate the discover of new therapeutic targets or help the insightful use of prior inflammatory targets for the prevention and treatment of venous thromboembolism.

## Figures and Tables

**Table 1:** Mechanisms involved in endothelial damage in sepsis.

*Mechanism*	*Pathway*
*Endothelial apoptosis*	LPS induces cell death. NETs produce cell death through the direct toxicity of histones and proteases.
*Endothelial denudation*	LPS provokes endothelial cell detachment from basal membrane/internal elastic lamina.
*Endothelial sensitization*	ANGPT-2 sensitizes endothelial cells to inflammatory cytokines and promotes leakage.
*Endothelial permeability*	Inflammatory cytokines increase the EC permeability.
*Alteration of endothelial* *histology*	LPS can induce nuclear vacuolization, cytoplasmic swelling and protrusion and cytoplasmic fragmentation.
*Suppression of endothelial* *anticoagulant receptors*	Inflammatory cytokines downregulate EPCR and thrombomodulin.
*Catecholamines-induced injury*	Elevated levels of noradrenaline cause glycocalyx disruption.
*Reduced sensitivity to* *catecholamines*	LPS reduces vessel relaxation mediated by ACh.
*Joint proteins internalization*	Inflammation induces VE-cadherin dislocation.

LPS, lipopolysaccharide; NET, neutrophil extracellular trap; ACh, acetylcholine; ANGPT-2, angiopoietin-2; EPCR, endothelial protein C receptor and ECs, endothelial cells.

**Table 2:** Coagulation laboratory tests.

*Function Evaluated*	*Laboratory Tests*
*Platelets*	Platelets count
*Coagulation*	Partial thromboplastin time (PT) and activated partial thromboplastin time (aPTT)
	Fibrinogen
*Anticoagulant markers*	Protein C and antithrombin III (AT III)
*Fibrinolysis markers*	D-dimer
*Fibrinolytic activity*	Plasminogen and α2-antiplasmin
*Antifibrinolytic activity*	Plasminogen activator inhibitor 1 (PAI-1)
*DIC markers*	Prothrombin activation fragment Fl and F2, factor IX (FIX), and factor X (FX) activation peptides

## Data Availability

Not applicable.
